# Meniscal Tears: Current Understanding, Diagnosis, and Management

**DOI:** 10.7759/cureus.8590

**Published:** 2020-06-13

**Authors:** Kavyansh Bhan

**Affiliations:** 1 Department of Trauma and Orthopaedics, Whipps Cross University Hospital, London, GBR

**Keywords:** meniscus tear, meniscus repair, meniscectomy, regenerative medicine therapies

## Abstract

From once being labelled as a functionless remain of leg muscle, extensive scientific investigations in recent decades have described the meniscus as one of the most crucial structures of the knee. The incidence of meniscal injuries is on the rise and can be attributed to the increased participation of youth in sporting activities. MRI continues to be the imaging modality of choice, and surgical management is the mainstay of treatment for meniscal tears. Arthroscopic partial meniscectomy (APM) is currently the most performed orthopedic procedure around the globe. However, recent studies have conclusively shown that outcomes after an APM are no better than the outcomes after a sham/placebo surgery. Meniscal repair is now being touted as a viable and effective alternative. Meniscal repair aims to achieve meniscal healing while completely avoiding the adverse effects of partial meniscectomy. Meniscal repairs have grown in popularity over the past three decades and have proved to be a much more efficient alternative to partial meniscectomy. It is now increasingly recommended to attempt meniscal repair in all repairable tears, especially in young and physically active patients. Partial Meniscal implants have also shown excellent outcomes in long-term studies, but its efficacy in acute settings still requires further research. Research performed on various techniques of meniscal regeneration looks promising, and regenerative medicine appears to be the way forward. This review aims to critically discuss the current understanding of the meniscus, its role in biomechanics of the knee joint, and the current methods used to diagnose and manage meniscal tears.

## Introduction and background

Though initially described as a functionless remain of a leg muscle [1], extensive scientific investigations in recent decades have described the meniscus as a vital part of the knee joint with anatomical, biomechanical, and functional importance [2]. The incidence of meniscal injuries is on the rise and can be partly attributed to increased participation in sports as well as the recent advances and easy availability of imaging technology such as MRI [3]. A conservative estimate pegs the incidence of meniscal tears at 60 per 100,000, though the true incidence is likely to be grossly underestimated [3]. The literature suggests that knees with known meniscal injury have accelerated cartilage wear, leading to an early onset osteoarthritis. A study by Jarraya et al. found that more than 75% of patients with symptomatic osteoarthritis have a meniscal injury [4]. In fact, meniscal injury is one of the most common sports injuries in day-to-day practice, and thus its prompt diagnosis and appropriate management have become an increasingly important part of orthopedic research.

Over the last four decades, management of meniscal injuries has seen great advances. Until the 1970s, total meniscectomy was the gold standard in the management of meniscal tears, owing to the then accepted notion of the meniscus being a functionless remnant vestige [1]. However, when postmeniscectomized knees were radiologically examined, it was found the postmeniscectomy knees had femoral condylar flattening, narrowing of joint space, and a predisposition to early degenerative changes [5]. It was suggested that the meniscus has an important weight-bearing function, and its absence interferes with the biomechanics of knee joint, leading to early degenerative changes. This led to the concept of meniscus preservation surgery. Over the years, preservation has shown a high success rate in terms of time to recovery and the functional outcome [6]. This has led to meniscus surgeries being one of the most common orthopedic operations performed, with incidence varying from 17 meniscus procedures per 100,000 population in the United States to 154 procedures per 100,000 in Korea [7]. This review aims to critically discuss the current understanding of the meniscus, its role in biomechanics of the knee joint, and the current methods used to diagnose and manage meniscal tears based on available evidence, and to look into its future potential.

Role of the meniscus

The meniscus is a relatively avascular structure with extremely limited blood supply. However, it is an indispensable part of biomechanical function of the knee. The meniscus is responsible for increasing the congruence of articulating surfaces of the knee joint [8]. Besides this, meniscus also plays a vital role in shock absorption and load transmission while walking and other activities [9]. Furthermore, it is also helpful in providing stability to the knee joint, limiting flexion and extension of the knee joint at extreme angles, and providing proprioception [10]. All these reasons may explain the increasing interest in meniscal injuries over the past few years.

Presentation

Meniscal tears can cause a range of symptoms, including pain localizing to the joint line, swelling, clicking, catching, locking, and the classic “giving away” of the knee. They are more commonly seen in men as compared to women, with up to 80% of all meniscal tears being reported in men [9]. Many patients have also reported waking up from sleep due to the pain. This can be explained by the possible scenario of a tender medial aspect of the knee colliding with the other knee while the patient rolls over in his sleep [11]. It is not uncommon to see meniscal injuries in conjunction with damage to structures such as anterior cruciate ligament (ACL), posterior cruciate ligament, or other bony injuries.

Diagnosis

**Figure 1:**
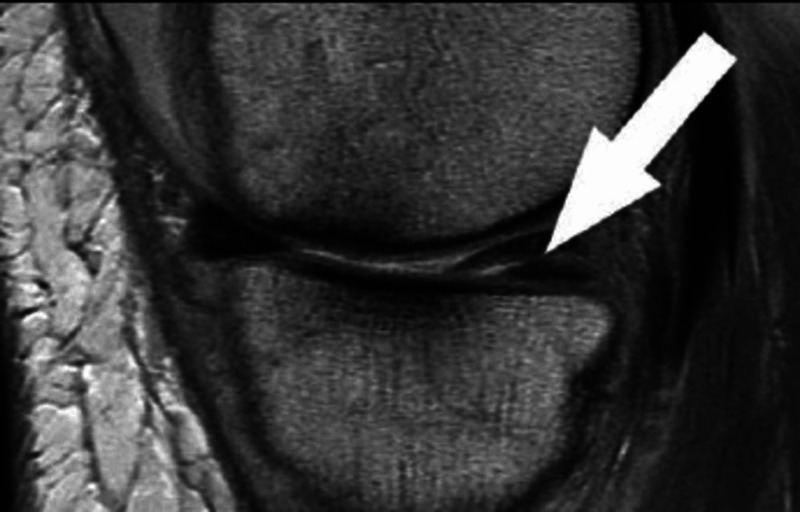
An example of a proton-weighted sagittal image showing a posterior horn medial meniscus horizontal tear (white arrow)

The onus lies on the orthopedic surgeon to effectively correlate clinical information, radiological images, and his/her clinical expertise to devise an individualized management plan for the meniscal tear. The severity of the symptom seldom corresponds to the type and location of the tear [12]. Detailed history-taking along with thorough clinical examination may not always clinch the diagnosis, and hence radiographic and arthroscopic evaluations should be conducted for confirmation of the diagnosis. Special clinical tests such as McMurray’s, Apley’s, and Thessaly’s tests may have been long recommended for diagnosing a tear, but their accuracy and reliability remain poor [13]. Plain radiographs are not recommended for routine evaluation of meniscus tears and are recommended in select conditions such as chondrocalcinosis [14]. MRI continues to be the imaging modality of choice, with sensitivity and specificity for diagnosing meniscus tears being as high as 93% and 88%, respectively [15]. On MRI, meniscal tears are usually diagnosed as a linear signal intensity that extends from meniscal substance to a free edge (Figure 1). Diagnostic arthroscopy without a therapeutic component is not recommended.

Treatment

With modern literature emphasizing the vital functions of the meniscus and possibility of early onset osteoarthritis in the absence of the meniscus, orthopedic surgeons have shifted their management goal from resection to preservation, repair, and reconstruction of the meniscus [12]. The treatment, however, still depends on several factors, namely age, comorbidities, symptoms, and type and location of tear. Conservative management is advocated in tears located in the high vascularity zones of the meniscus, namely red/red zone or peripheral 30% of the medial meniscus and 25% of the lateral meniscus [16]. Higher success is seen if the said tears measure <5 mm, as they are considered to be stable tears [16].

Surgical management is the mainstay of treatment for all other types of meniscal tears. Until 1960, open meniscectomy was the standard surgical treatment for meniscal tears [17]. Ikeuchi performed the first arthroscopic repair in 1969, and since then various arthroscopic techniques have evolved [18].

Since total meniscectomy can directly lead to a rise in contact stress, an accelerated onset of osteoarthritis, and even symptomatic varus deformities in the older age group, it is now considered to be an almost obsolete treatment option [19]. Arthroscopic partial meniscectomy (APM) is currently the most performed orthopedic procedure around the globe. However, recent studies have conclusively shown that outcomes after an APM are no better than the outcomes after a sham/placebo surgery [20]. It has been termed by many researchers as a “useless” surgery, and recent clinical guidelines have been increasingly recommending against the procedure [21].

Meniscal repair is now being touted as a viable and effective alternative. Meniscal repair aims to achieve meniscal healing while completely avoiding the adverse effects of partial and total meniscectomy. While short-term outcomes of meniscal repairs have been fairly successful, with a failure rate of less than 10% at two-year follow-ups [22], the long-term results have not been encouraging and failure rates of up to 30% have been reported at five-year follow-ups [23]. Despite various techniques being developed, such as the inside-out technique, meniscal fixators, all-inside technique, and outside-in technique, failure rates at long-term follow-ups have remained fairly consistent, ranging from 23% to 30%.

Other treatment options include meniscal allografting. Although a complex procedure, 10-year follow-up survival stood at a promising 89.2% [24]. Less complex and minimally invasive procedures such as meniscal scaffolds have also been recently approved by the FDA. Scaffolds are available off the shelf and are designed to allow in-growth of tissue on to the scaffold to mimic physiological replacement [25]. Another promising option is a partial meniscal substitute, which is designed to re-establish load distribution across the knee joint, thereby providing a chondroprotective property.

## Review

Over the years, the role of MRI has become indispensable in diagnosing not only meniscal injuries but also almost all the intra-articular pathologies of the knee [26]. Studies have also shown that trained radiologists have a much higher specificity and sensitivity for diagnosing meniscal tears [26]. With clinical examinations having low accuracy in diagnosis, MRI has become the gold standard investigation for meniscal tears. However, owing to its non-invasive nature and high reliability index, MRI is being excessively “medicalized” and being routinely advised in cases where confirmation with an MRI is deemed unnecessary. For instance, patients already diagnosed with severe osteoarthritis on plain X-rays and exhibiting meniscal mechanical symptoms can be safely assumed to have a meniscal tear, and an MRI in such situations does not necessarily change the course of treatment, which would usually be medical management [27].

Surgical management of meniscal injuries has taken rapid strides in recent times. However, the role of the commonest surgery performed worldwide for meniscal tears, APM, remains controversial. Studies have suggested that the rate of cartilage loss can be as high as 7% per year for healthy young individuals undergoing partial meniscectomy early on in their life [28]. Following an APM, unfavorable outcomes have been reported in as many as 30% of the cases at a 10-year follow-up [29]. However, due to the easier learning curve and reportedly satisfactory short-term outcomes along with pain reduction at two-year follow-up, APM continues to be one of the most common orthopedic operations being performed worldwide [30].

Over the last two to three decades, great emphasis has been laid on meniscal repair and preservation. The aim is to preserve as much of the meniscus as possible and to avoid meniscectomy. To achieve this, the repair techniques have evolved substantially, with open meniscectomy becoming almost obsolete and arthroscopic techniques being developed consistently. Various arthroscopic techniques such as outside-in, inside-out, and all-inside have been extensively published, with all-inside and inside-out techniques being the most preferred by orthopedic surgeons around the globe [23]. The success of repair, however, depends on a range of factors, most importantly the location and type of the tear. The medial meniscus has its blood supply concentrated in the peripheral 20-30%, whereas the lateral meniscus is perforated by vessels in 10-25% of its periphery. The remainder 70-75% of both menisci receive their nutrition through diffusion in the absence of any direct arterial supply [31]. This has led to the classification of meniscal tears based on their relative location to the blood supply, building on the zonal classification of meniscal tears by Anderson et al. [32]. Tears located at the periphery or zone 1 are referred to as red-red (R/R) tears, tears located in the middle third or zone 2 are referred to as red-white (R/W) tears, and tears located in the inner third or zone 3 are referred to as white-white (W/W) tears [33]. The tears in zones with better blood supply, namely R/R and R/W, have significantly improved outcomes as opposed to tears in the W/W zone [31]. The morphology of the tears also play an important role in the healing, with horizontal or vertical tears having a success rate of up to 85% after surgery [34]. The systematic review by Nepple et al. also highlighted the possibility of lateral meniscus tears responding more favorably to repair as opposed to medial meniscus tears [23]. Re-tear rate for medial meniscus tears following a repair has been shown to be as high as 36.4% [35].

Various studies have compared the outcomes of partial meniscectomy with meniscal repairs. While meniscectomy has better pain relief as opposed to repair in the early stages, it can be attributed to the fact that a meniscectomy removes the pathology immediately by excision of the tear, whereas repairs are based on the principle of regeneration and hence have better outcomes at long-term follow-up [36]. Higher activity levels and better functional outcomes are seen with meniscal repair as opposed to meniscectomy, as it postulated that preservation of the meniscus leads to higher stability of the knees and delays the onset of osteoarthritis. Recent studies have shown that repairs exhibit improved healing in knees where ACL is also repaired along with the meniscus [36]. Although no consensus exists on why this happens, various theories have been put forward. Some researchers argue that an ACL reconstruction results in excessive intraarticular trauma to the joint, thereby leading to additional bleeding and fibrin clot formation, which, in turn, means higher availability of growth factors [37]. Another possible explanation is the much slower rehabilitation protocols followed after an ACL reconstruction, thereby producing a low force and more secure environment for meniscal healing [38]. Due to these reasons, meniscal repair is now being encouraged wherever possible.

In conditions wherein repair is no longer a possibility or when there has already been a total or partial meniscectomy performed, meniscus replacement has proved its worth [39]. A replacement may be possible through meniscal allograft transplantation (MAT), whereby the whole meniscus is replaced with or without bone plugs. The risk of disease transmission inherently associated with allografts can be significantly reduced by freezing the graft at -180 degree Celsius with the addition of anti-freezing agents such as glycerol, a technique called cryopreservation [40]. Partial replacement of the meniscus has been achieved by means of meniscal scaffolds. While scaffolds may attempt regeneration of the native meniscus, studies have shown that the tissue grown is different from the native meniscus of the patient [39]. However, a recent systematic review on clinical outcomes of meniscal scaffold reported a failure rate of only 6.7% to 9.9% at 45 months’ follow-up [41]. An improvement in functional outcomes assessed using Lysholm Knee Score and Tegner Activity Score meant an improvement in the functional activity postmeniscal scaffold replacement [42]. However, despite the promising results, high-quality long-term follow-up studies are required to accurately assess the efficiency of meniscal scaffolds.

A lot of focus has been placed on regenerating the meniscus, and this has propelled further research in regenerative medicine. The use of growth factor for meniscal healing is under development to allow selective control of cell activity in order to promote healing [39]. Another clinical study advocating the use of mesenchymal stem cells in the form of an injection into the knee has shown a considerable increase in the volume of the meniscus at two-year follow-up [43]. Guo et al. have listed the different types of scaffolds currently under development, including absorbable scaffolds, hydrogel scaffolds, and three-dimensional printed scaffolds [44]. These are all exciting concepts that are currently being worked upon.

## Conclusions

Meniscal tears, although a common orthopedic pathology, can be a challenge to treat. Diagnosis of meniscal injuries is not only dependent on good history taking and clinical examination but also almost inevitably requires confirmation with MRI. A thorough understanding of the unique anatomical structure and the knowledge of vascularity along with the zonal classification of tears are extremely important for designing an effective management plan. While conservative management has its role and can be indicated in cases with advanced osteoarthritis or in patients with small tears, partial meniscectomy continues to be the most performed procedure for meniscal injuries. While its efficacy is a matter of debate, its short learning curve and acceptable short-term results have deterred many orthopedic surgeons to switch to more effective procedures. Meniscal repairs have grown in popularity over the past three decades and have proved to be a much more efficient alternative to partial meniscectomy. It is now increasingly recommended to attempt meniscal repair in all repairable tears, especially in young and physically active patients. Partial meniscal implants have also shown excellent outcomes in long-term studies, but their efficacy in acute settings still requires further research. Research performed on various techniques of meniscal regeneration looks promising, and regenerative medicine appears to be the way forward.
